# A cell factory of *Bacillus subtilis *engineered for the simple bioconversion of *myo*-inositol to *scyllo*-inositol, a potential therapeutic agent for Alzheimer's disease

**DOI:** 10.1186/1475-2859-10-69

**Published:** 2011-09-07

**Authors:** Masaru Yamaoka, Shin Osawa, Tetsuro Morinaga, Shinji Takenaka, Ken-ichi Yoshida

**Affiliations:** 1 Department of Agrobioscience, Graduate School of Agricultural Science, Kobe University, 1-1 Rokkodai, Nada, Kobe 657 8501, Japan; 2 Process Engineering Department, Manufacturing Management Division, Sysmex Corporation, 4-4-4 Takatsukadai, Nishi-ku, Kobe 651-2271, Japan

## Abstract

**Background:**

A stereoisomer of inositol, *scyllo*-inositol, is known as a promising therapeutic agent for Alzheimer's disease, since it prevents the accumulation of beta-amyloid deposits, a hallmark of the disease. However, this compound is relatively rare in nature, whereas another stereoisomer of inositol, *myo*-inositol, is abundantly available.

**Results:**

*Bacillus subtilis *possesses a unique inositol metabolism involving both stereoisomers. We manipulated the inositol metabolism in *B. subtilis *to permit the possible bioconversion from *myo*-inositol to *scyllo*-inositol. Within 48 h of cultivation, the engineered strain was able to convert almost half of 10 g/L *myo*-inositol to *scyllo*-inositol that accumulated in the culture medium.

**Conclusions:**

The engineered *B. subtilis *serves as a prototype of cell factory enabling a novel and inexpensive supply of *scyllo*-inositol.

## Background

Alzheimer's disease is one of the most common and problematic forms of dementia [1]. In 2006, 26.6 million people suffered from Alzheimer's disease worldwide, and by 2050, the disease is predicted to increase, affecting 1 in every 85 individuals [2]. Hundreds of clinical trials have been conducted to identify possible treatment strategies for the disease, but despite these efforts no promising strategy has been established. At present, most of the treatment strategies currently used offer only a small symptomatic benefit, and in fact no treatment is available to stop the progression of the disease.

**Figure 1:**
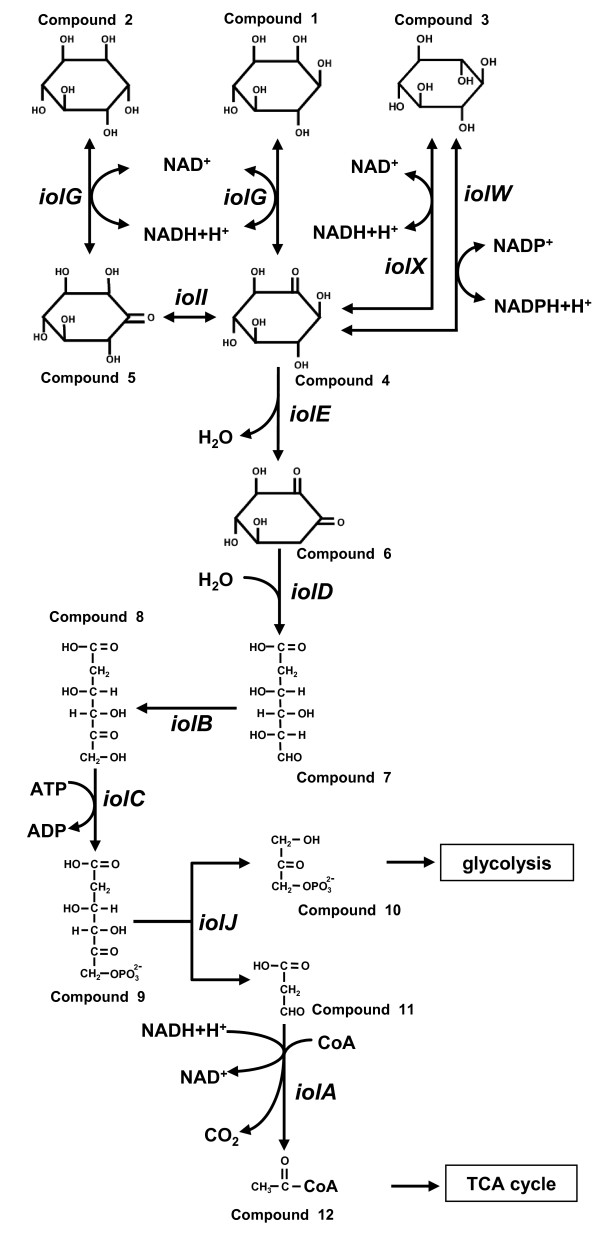
**Inositol metabolism in *B. subtilis***. *B. subtilis iol *genes encoding enzymes for reactions in the inositol catabolic pathway and the respective intermediate compounds are shown. The intermediate compounds are *myo*-inositol (compound 1), D-*chiro*-inositol (compound 2), *scyllo*-inositol I (compound 3), *scyllo*-inosose (compound 4), 1-keto-D-*chiro*-inositol (compound 5), 3D-(3,5/4)-trihydroxycyclohexane-1,2-dione (compound 6), 5-deoxy-D-glucuronic acid (compound 7), 2-deoxy-5-keto-D-gluconic acid (compound 8), 2-deoxy-5-keto-D-gluconic acid 6-phosphate (compound 9), dihydroxyacetone phosphate (compound 10), malonic semialdehyde (compound 11), and acetyl-CoA (compound 12).

Inositol (1,2,3,4,5,6-cyclohexanehexol) has nine possible stereoisomers due to epimerization of the six hydroxyl groups. The most abundant stereoisomer in nature is *myo*-inositol (compound 1, Figure 1), which is the structural basis for a number of secondary messengers in eukaryotic cells [3]. In plants, *myo*-inositol hexakisphosphate or phytic acid often occurs in high bran cereals, serving as the principal storage form of phosphate. This is the main industrial source of *myo*-inositol [4]. The other inositol stereoisomers are relatively rare in nature but some exert specific health-promoting activities. D-*chiro*-inositol (compound 2) is beneficial for patients with hyperglycemia and polycystic ovary syndrome since it restores insulin sensitivity [5] and recovers normal ovulation [6], respectively. Accumulating evidence indicates that another isomer, *scyllo*-inositol (compound 3), has exceptional potential as a therapeutic agent for Alzheimer's disease [7].

*scyllo*-Inositol is sometimes found in plants and also occurs naturally in human brain as a minor substance that readily crosses the blood-brain barrier [8]. Aggregation of the beta-amyloid peptide in brain cells is a key pathological feature of Alzheimer's disease [9]. *scyllo*-Inositol directly interacts with the beta-amyloid peptide and blocks the development of fibers, alleviating memory deficits and other symptoms associated with beta-amyloid accumulation [10,11]. Furthermore, mice fed with *scyllo*-inositol have been demonstrated to have decreased disease symptoms with better cognitive function and greater longevity because the amyloid plaques disappeared and inflammation declined. *scyllo*-Inositol confers these benefits not only in very young disease-free mice but also in diseased mice [7]. Thus, *scyllo*-inositol got fast-track designation from the US Food and Drug Administration for treatment of mild to moderate Alzheimer's disease, and was granted a US patent in 2009 [12]. Phase 2 clinical trials of *scyllo*-inositol treatment have already been conducted with positive results, and plans for Phase 3 trials have been announced [13].

*Bacillus subtilis *is one of the best studied Gram-positive bacteria, and its unique inositol metabolism involves at least three of the stereoisomers, *myo*-inositol, *scyllo*-inositol, and D-*chiro*-inositol (Figure 1) [14]. The *B. subtilis iolABCDEFGHIJ *operon encodes enzymes involved in multiple steps of inositol metabolism [15]. In the first step, *myo*-inositol is converted to *scyllo*-inosose (compound 4, Figure 1) by *myo*-inositol dehydrogenase, IolG, with NAD^+ ^reduction. IolG also dehydrogenates D-*chiro*-inositol to 1-keto-D-*chiro*-inositol (compound 5), which is subsequently isomerized by IolI to *scyllo*-inosose [16]. In the second step, *scyllo*-inosose is dehydrated by IolE to 3D-(3,5/4)-trihydroxycyclohexane-1,2-dione (compound 6) [17]. In the third step, IolD catalyzes the hydrolysis of 3D-(3,5/4)-trihydroxycyclohexane-1,2-dione to yield 5-deoxy-D-glucuronic acid (compound 7). In the next step, IolB catalyzes the isomerization of 5-deoxy-D-glucuronic acid to 2-deoxy-5-keto-D-gluconic acid (compound 8), which is subsequently phosphorylated by IolC kinase to 2-deoxy-5-keto-D-gluconic acid 6-phosphate (compound 9) [18]. 2-Deoxy-5-keto-D-gluconic acid 6-phosphate is an intermediate that acts as an inducer by antagonizing DNA binding of IoIR, a repressor controlling transcription of the *iol *operon [19]. Finally, IolJ aldolase catalyzes the conversion of 2-deoxy-5-keto-D-gluconic acid 6-phosphate to dihydroxyacetone phosphate (compound 10) and malonic semialdehyde (compound 11). The former is a glycolytic intermediate and the latter is converted to acetyl-CoA (compound 12), which enters the tricarboxylic acid cycle, and CO_2 _by the IolA reaction.

*B. subtilis *possesses two additional and distinct inositol dehydrogenases, IolX and IolW, which specifically act on *scyllo*-inositol with NAD^+ ^and NADP^+ ^reduction, respectively [14]. Each of these enzymes convert *scyllo*-inositol to *scyllo*-inosose, which is the same compound produced from *myo*-inositol by IolG and is readily degraded further via the metabolic pathway described above [18]. *iolX *transcription is induced after the addition of *scyllo*-inositol to the growth medium of cells, and its inactivation inhibits cell growth in the presence of *scyllo*-inositol as a carbon source, indicating that IolX could function physiologically as a catabolic enzyme [14]. In contrast, *iolW *transcription is constitutive and its inactivation does not alter the cell growth in the presence of *scyllo*-inositol. Our previous study suggested that IolW may generate *scyllo*-inositol from *scyllo*-inosose with NADPH oxidation, although its physiological role remains unidentified [14].

In this study, we manipulated the inositol metabolism in *B. subtilis *to control the interconversion among inositol stereoisomers secreted into the culture medium, permitting a simple and inexpensive bioconversion of *myo*-inositol to *scyllo*-inositol. The engineered *B. subtilis *materialized our cell factory concept for *scyllo*-inositol production.

## Results and Discussion

### Analyzing mutations for efficient bioconversion of *myo*-inositol to *scyllo*-inositol

Previously we demonstrated that *B. subtilis *strain YF256 (Table 1) was capable of bioconverting *myo*-inositol to D-*chiro*-inositol [16]. Because of *iolR *inactivation [19], strain YF256 constitutively expresses *iolG*, *iolE41*, and *iolI*. The missense mutation allele *iolE41 *abolishes IolE dehydratase activity, resulting in the intracellular accumulation of *scyllo*-inosose from *myo*-inositol due to the IolG reaction [16]. *scyllo*-Inosose is isomerized by IolI to 1-keto-D-*chiro*-inositol and subsequently reduced by IolG to D-*chiro*-inositol; thus, *myo*-inositol is converted to D-*chiro*-inositol, which then appears in the inositol bioconversion medium [16].

**Table 1:** Bacterial strains used in this study.

Strain	Relevant genotype	Source or reference
60015	Wild-type	Laboratory stock
BFS3018	*iolX*::pMutin2(*erm*)	National Institute of Genetics [14]
FU341	*asnH*::*spc*	[24]
TM038	*iolE41 iolI*::*spc iolR*::*cat*	This work
TM039	*iolE41 iolI*::*spc iolR*::*cat iolX*::pMutin4(*erm*)	This work
TM040	*iolE41 iolI*::*spc iolR*::*cat iolW*::pMutin2(*erm*::*tet*)	This work
TM041	*iolE41 iolI*::*spc iolR*::*cat iolW*::pMutin2(*erm*::*tet*) *iolX*::pMutin4(*erm*)	This work
TM043	*iolW*::pMutin2(*erm*::*tet*)	[14]
YF256	*iolE41 iolR*::*cat*	[17]

The integration vector pMutin2 originally possesses the *erm *marker [26], which was replaced with the *tet *marker in TM043 as described previously [23].

**Figure 2:**
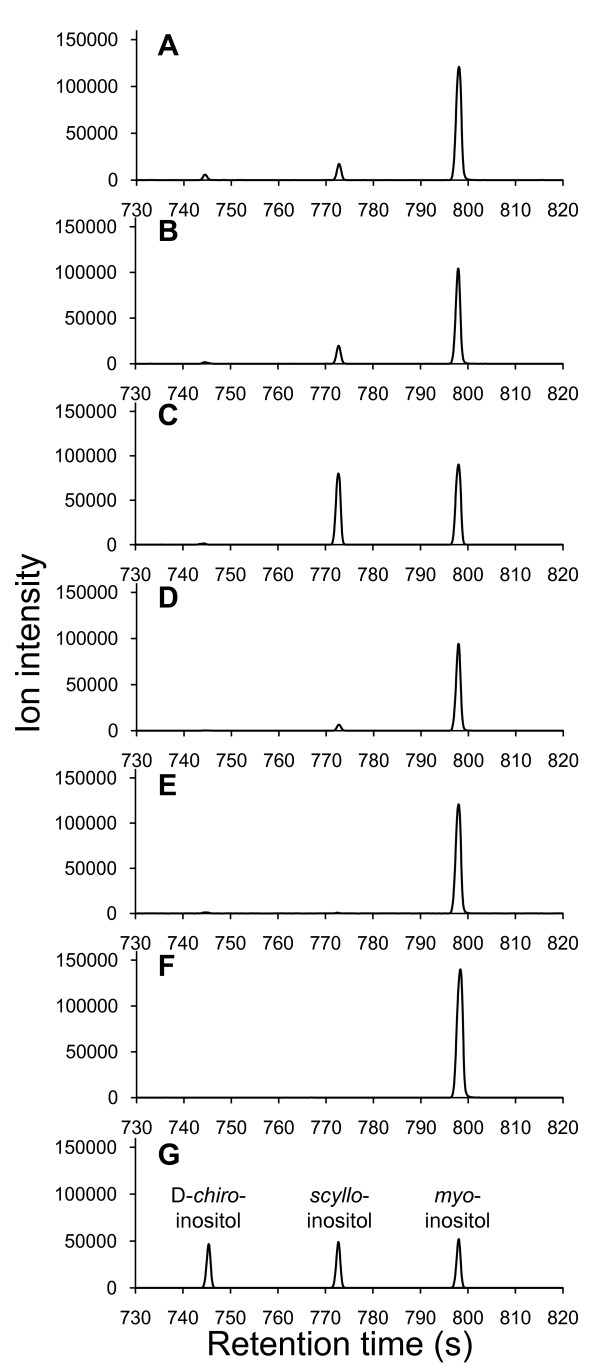
**Inositol stereoisomers present in the medium**. Strains of *B. subtilis*, including YF256 (A), TM038 (B), TM039 (C), TM040 (D), or TM041 (E), were grown in the inositol bioconversion medium containing 2% Bacto soytone. At the indicated time points, aliquots of the culture medium were subjected to GC-TOFMS analysis as described previously [20] in parallel with the initial medium (F) and authentic standards as indicated (G). The experiments were repeated independently at least three times with similar results, and the representative data are shown.

In this study, we re-examined the medium in which strain YF256 was cultured for 24 h by an improved GC-TOFMS system [20]. We were surprised to detect not only D-*chiro*-inositol but also *scyllo*-inositol (Figure 2A), thus revealing that our previous high-performance liquid chromatography analysis failed to detect *scyllo*-inositol [16]. Furthermore, this result suggested that some of the accumulated *scyllo*-inosose could be converted to *scyllo*-inositol, probably involving IolX and/or IolW.

When *iolI *was inactivated (strain TM038, Figure 2B), conversion of *scyllo*-inosose to D-*chiro*-inositol decreased to a negligible level, whereas that of *scyllo*-inositol increased; however only slightly. These results indicated that we could eliminate the metabolic pathway for D-*chiro*-inositol by inactivating *iolI*, although this was not sufficient to increase the conversion to *scyllo*-inositol. Therefore, *iolX *and/or *IolW *were inactivated to examine their involvement in *scyllo*-inositol production. When *iolW *was functional, *iolX *inactivation alone drastically elevated the concentration of *scyllo*-inositol present in the medium (strain TM039, Figure 2C), suggesting that IolX may degrade *scyllo*-inositol. In contrast, IolW was essential for efficient *scyllo*-inositol production (strains TM040 and TM041, Figure 2D and 2E, respectively). These results clearly indicate that IolW is crucial for the conversion of *scyllo*-inosose to *scyllo*-inositol.

### Properties of the *scyllo*-inositol producing *B. subtilis *cell factory

**Figure 3:**
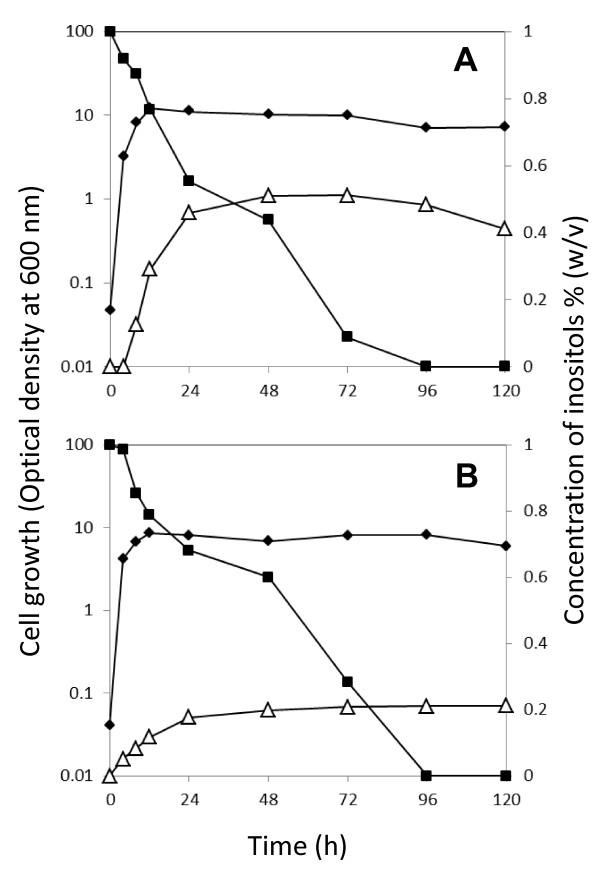
**Time course of *scyllo*-inositol production and *myo*-inositol consumption during inositol bioconversion along with the cell growth**. The bioconversion from *myo*-inositol to *scyllo*-inositol was performed in the media containing 2% (A) or 1% (B) Bacto soytone. The data presented are optical density of the cells (black diamonds), and concentration of *myo*-inositol (black squares) and *scyllo*-inositol (white triangles). The experiments were repeated independently at least three times with similar results, and the representative data sets are shown.

After 48-h culture of strain TM039 in the inositol bioconversion medium (Figure 3A), almost half of the *myo*-inositol was converted to *scyllo*-inositol. This simple bioconversion was so successful that it realized our concept of cell factory to produce *scyllo*-inositol with higher translational potential in the yield and purity of product than the other methods previously described [21,22]. Further culture resulted in the consumption of not only *myo*-inositol but also some of the *scyllo*-inositol, and particularly *myo*-inositol was consumed almost completely after 96 h of culture whereas *scyllo*-inositol remained relatively intact. The results implied that the mutated IolE41 enzyme, which did not support cell growth using *myo*-inositol as the carbon source [17], might possibly retain a certain lower level of *scyllo*-inosose degrading activity, which resulted in the decrease in the two inositol isomers; because *myo*-inositol is preferentially converted to *scyllo*-inosose by IolG reaction, it decreased more obviously. Although we do not have experimental evidences to prove the possibility mentioned above, nevertheless the selective consumption of *myo*-inositol could be even practical for a cell factory making the *scyllo*-inositol product free from significant *myo*-inositol contamination. However, to avoid wastage of both the material and the product, possible strategies to stop the inositol degradation will be considered, including in-frame deletion of *iolE*.

In principal, we could assume that IolG reaction is due to a lowering of the intracellular NAD^+^/NADH ratio, whereas the IolW reaction elevates the NADP^+^/NADPH ratio (Figure 1). If this is actually the case, the inevitable problem of cofactor imbalance must have been induced in the cells. Although considerably efficient bioconversion has already been achieved (Figure 3A), it may be improved by correcting the possible cofactor imbalance under the given conditions. Interestingly, when the major nutrient (Bacto soytone) contained in the conversion medium was reduced by half, no severe effect on the cell growth was observed, nevertheless only *scyllo*-inositol production was impaired significantly while *myo*-inositol was consumed almost completely (Figure 3B). The results imply that there might be some nutritional and/or metabolic factors limiting the bioconversion, which should be identified and then controlled in the next generation of realistic cell factory with optimized efficiency in *scyllo*-inositol production.

It was demonstrated that *B. subtilis *possesses two distinct inositol transporters, IolT and IolF, which are different in substrate specificity to *myo*-inositol and D-*chiro*-inositol [23]. Our preliminary observations suggested that *scyllo*-inositol transport into the cells could mainly depend on IolT, which is similar to *myo*-inositol (data not shown), and overexpression of *iolT *may not be effective for improving the conversion efficiency. However, it has not yet been explained how the inositol stereoisomers are secreted into the medium after conversion; hence, we speculate the involvement of an unknown efflux pump, which is also currently being investigated as one of the possible targets to be engineered.

## Conclusions

*B. subtilis *is one of the best studied gram-positive bacteria. Its unique inositol metabolism has been completely elucidated to involve at least three inositol stereoisomers, including *scyllo*-inositol and *myo*-inositol. Our previous knowledge and findings suggested possible interconversion among the stereoisomers. Therefore, we manipulated the metabolic pathway to realize a cell factory concept enabling the simple and inexpensive bioconversion of *myo*-inositol to *scyllo*-inositol, which is a promising therapeutic agent for Alzheimer's disease.

## Methods

### Bacterial strains and their constructions

Bacterial strains used in this study are listed in Table 1. The *iolI *gene of strain YF256 was inactivated by inserting a spectinomycin resistance gene (*spc*) cassette as follows. A DNA fragment carrying the *spc *cassette was amplified from strain FU341 DNA [24] by PCR using IolI3F and IolI4R primer pair (Table 2). Further, two DNA fragments corresponding to the upstream and downstream regions of *iolI *were amplified from strain 60015 DNA by PCR using IolI1F/IolI2R and IolI5F/Iol6R primer pairs, respectively, (Table 2). The 5' end sequences of IolI3F and Iol4R were complementary to IolI2R and IolI5F, respectively. The three PCR fragments were mixed and ligated using PCR performed with the outside primer pair IolIF1/Iol6R. The resulting PCR fragment was used to transform strain YF256 to obtain strain TM038 with spectinomycin resistance (*iolI*::*spc*; Table 1).

**Table 2:** Oligonucleotide primers used in this study

Primer	Sequence
iolI1F	5'-TGCGGTTGAACTTGAAGTGG-3'
iolI2R	5'-TCTTCTGCTCTGTCACAAGC-3'
iolI3F	5'-GCTTGTGACAGAGCAGAAGACAATAACGCTATTGGGAG-3'
iolI4R	5'-GAACCCATTGCATGGAAGTGCTATATGCTCCTTCTGGC-3'
iolI5F	5'-CACTTCCATGCAATGGGTTC-3'
iolI6R	5'-ATATTGATCTTCGCGTGGCC-3'

Strain TM038 was transformed with strain BFS3018 DNA [14] to obtain strain TM039 with erythromycin resistance (*iolX*::pMutin4(*erm*); Table 1) and with strain TM043 DNA [14] to obtain strain TM040 with tetracycline resistance (*iolW*::pMutin2(*erm*::*tet*); Table 1). Strain TM040 was further transformed with strain BFS3018 DNA to obtain strain TM041 with erythromycin resistance (*iolW*::pMutin2(*erm*::*tet*) *iolX*::pMutin4(*erm*); Table 1).

### Culture conditions

Bacterial strains were maintained in Luria-Bertani medium [25]. For inositol bioconversion, a 500-ml Sakaguchi flask with a breathable foam stopper, containing 30 ml of inositol bioconversion medium, containing 2% or 1% (w/v) Bacto soytone (Becton, Dickinson and Co., Sparks, MD), 0.5% Bacto yeast extract (Becton, Dickinson and Co.), 0.5% NaCl, and 1% *myo*-inositol, was inoculated with one of the *B. subtilis *strains at an optical density of 0.05 at 600 nm, and incubated at 37°C with shaking at 150 rpm.

### Measurement of inositol stereoisomers in the medium

After bioconversion, aliquots of the culture medium were subjected to gas chromatography-time-of-flight mass spectrometry (GC-TOFMS) analysis as described [20] but with some modifications as follows. After removing bacterial cells by centrifugation, an aliquot of the culture medium was evaporated in a test tube under vacuum. The dried pellet was dissolved in the extraction mixture of water, chloroform, and methanol (2:2:5), and incubated at 150°C for 30 min with vigorous shaking. After centrifugation at 40°C for 3 min at 16,000 g, the supernatant was transferred to another tube and diluted appropriately with pure water. After another centrifugation, part of the supernatant was dried completely in another tube. The dried pellet was dissolved, and the substances present in it were derivatized with methoxyamine hydrochloride in pyridine at 30°C for 90 min with vigorous shaking. After the addition of N-methyl-N-(trimethylsilyl) trifluoroacetamide (GL Science, Tokyo, Japan), the derivatized substances were further incubated at 37°C for 30 min, and then injected via an Agilent 7683B autosampler into an Agilent 7890A GC (Agilent Technologies, Palo Alto, CA) coupled to a Pegasus HT TOFMS (Leco Corp., St Joseph, MI) with the settings as described [20]. Peak deconvolution identification and quantification were performed using the Pegasus ChromaTOF software package ver. 4.21 (Leco Corp.). Commercially available authentic inositols were derivatized and analyzed in parallel with the experimental samples. The mass spectra and retention time obtained were used to identify the stereoisomers, and ion intensities were used to calculate concentration of the stereoisomers.

## Competing interests

The content of this manuscript is relevant to a patent application made by Kobe University (PCT/JP2009/005782), but all authors declare that they have no competing interests.

## Authors' contributions

All authors read and approved the manuscript. MY, SO, and TM equally contributed to experiments. ST was involved in data analysis and interpretation. KY designed and conducted this research project as the principal investigator, being also responsible for manuscript preparation.
